# Cation Chemistry and Molecular Weight Effects on the Ion Conductivity in PEO-based Electrolytes

**DOI:** 10.1021/acsmacrolett.4c00802

**Published:** 2025-02-10

**Authors:** Chrysostomos Papamichail, Olympia Techlemtzi, Georgia Nikolakakou, Emmanouil Glynos

**Affiliations:** † Department of Materials Science and Engineering, University of Crete, P.O. Box 2208, 710 03, Heraklion, Crete, Greece; ‡ Institute of Electronic Structure and Laser, Foundation for Research and Technology-Hellas, P.O. Box 1385, 711 10 Heraklion, Crete, Greece; § Department of Chemistry, University of Crete, P.O. Box 2208, 710 03 Heraklion, Crete, Greece

## Abstract

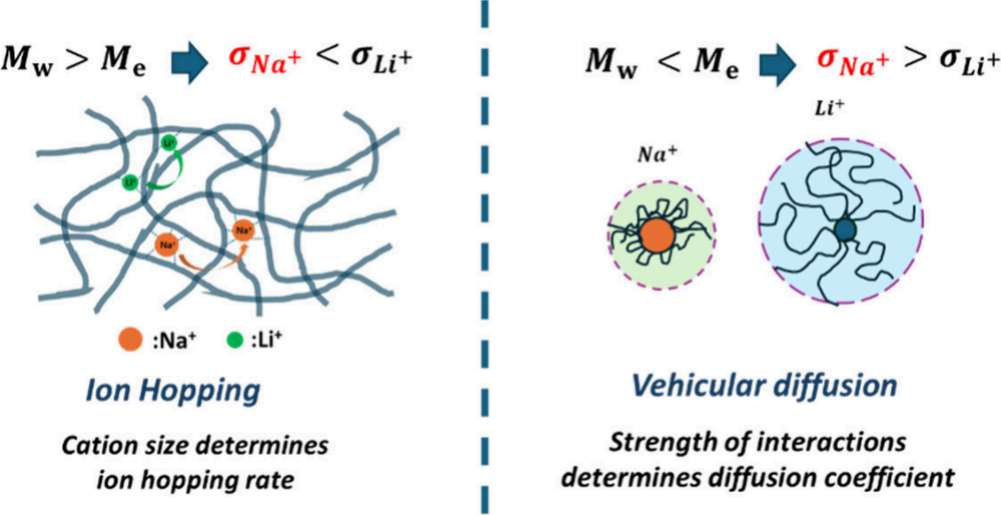

This study investigates the fundamental influence of cation chemistry on the ionic conductivity of PEO-based electrolytes, with implications for advancing polymer electrolyte design. Two PEO systems—high molecular weight (*M*_w_ = 100 kg/mol) and low molecular weight (*M*_w_ = 0.35 kg/mol)—were blended with LiTFSI and NaTFSI salts to explore ion transport mechanisms. In the high-*M*_w_ PEO, where ion hopping dominates, smaller Li^+^ ions exhibit higher conductivity (σ_LiTFSI_ > σ_NaTFSI_). In contrast, the low-*M*_w_ PEO, where ion diffusion is the primary mechanism, shows higher conductivity for larger Na^+^ ions (σ_NaTFSI_ > σ_LiTFSI_). In the former, rheology measurements indicate that larger Na^+^ cations form more transient EO:Na^+^ contact, hindering cation hopping and reducing conductivity. In the latter, the stronger EO:Li^+^ interactions lead to a larger hydrodynamic radius and slower diffusion. Notably, PEO-0.35K:NaTFSI exhibits a room-temperature conductivity of σ_NaTFSI_ ≈ 4 × 10^–4^ S/cm, meeting the requirements for practical applications. These findings highlight the potential of low-*M*_w_ PEO and Na-based electrolytes for the development of efficient Na-ion batteries.

The currently used lithium-ion batteries (LIBs), which rely on graphite anodes and layered oxide cathodes, are reaching their performance limits. As environmental, supply, and safety challenges emerge, the development of improvements and alternatives to LIBs has become increasingly important. Advanced lithium-ion technology focuses on replacing conventional graphite anodes with lithium-metal or silicon anodes, which can significantly enhance the energy density. Given lithium’s high power-to-weight ratio, lithium-based batteries are expected to maintain their dominant position, playing an increasingly critical role in electric transportation through the second half of this decade.^1−4^ However, environmental concerns and high costs limit the broader commercial adoption of lithium based batteries.^5,6^ These challenges arise primarily from the limited global reserves and uneven distribution of lithium resources, with about 70% concentrated in South America.^7^ Since 1991, the cost of lithium has nearly doubled, further complicating the economic viability of commercial applications.^8^ Additionally, the limited availability of lithium poses significant constraints on its use in large-scale energy storage systems.

Polymer electrolytes have attracted considerable attention due to their potential to transform energy storage and advanced technologies.^9−12^ In applications such as lithium-ion and solid-state batteries, they offer enhanced safety by reducing flammability and improving thermal stability, addressing significant concerns associated with traditional liquid electrolytes that are a mixture of a polar organic solvent and an alkali salt. Researchers are working to enhance the ionic conductivity at room temperature, a key factor for practical performance. Additionally, polymer electrolytes contribute to sustainability efforts, with ongoing developments in eco-friendly, renewable materials that reduce environmental impact.^13^ Beyond batteries, they play essential roles in fuel cells, supercapacitors, and electrochemical sensors, expanding their technological impact.^14^ Overcoming challenges such as conductivity enhancement and cost reduction could unlock groundbreaking advancements across multiple industries, making polymer electrolytes a cornerstone of future innovations.

The first discovery of ionic conductivity in lithium salt complexes with linear poly(ethylene oxide) (PEO) was reported in 1973 in *Polymer*, and its application in battery cells was demonstrated a few years later.^15^ Since then, PEO has been extensively studied due to its ability to solvate various salts with different cations. The ethylene oxide (EO) units strongly interact with cations, while PEO’s flexible chains and low glass transition temperature (*T*_g_) facilitate rapid ion transport. It is generally accepted that ion conduction occurs in the amorphous phase of PEO and is closely linked to its segmental dynamics.^10^ At low molecular weights (*M*_w_), unentangled linear PEO homopolymer electrolytes enable ion diffusion through the movement of the entire PEO chain, carrying the coordinated ion along.^16−20^ This mechanism is very efficient at room temperature, resulting in conductivities exceeding 10^–4^ S/cm, i.e., values close to those required for practical applications. Consequently, low *M*_w_PEO electrolytes have been successfully utilized as the conductive phase in all polymer nanostructured solid electrolytes.^21−23^ As the PEO molecular weight increases, polymer chains begin to form entanglements. This shifts ion diffusion to rely primarily on segmental dynamics and ion transport is mediated by Li^+^ hopping between neighboring ether oxygen atoms, which involves the breaking and forming of lithium–oxygen (Li–O) bonds—also known as fluctuation-driven diffusion.^17^ Both diffusion mechanisms are present in polymer systems, though their significance varies based on molecular weight. Consequently, at molecular weights above the molecular weight for entanglements (*M*_w_*>M*_e_≈1–2 kg/mol), the ionic conductivity no longer depends on the length of the PEO chain. In this regime the glass transition temperature, and corresponding segmental dynamics, is independent of *M*_w_. Ion hopping is slow at room temperature, resulting in low ion conductivity at room temperature in entangled systems (σ < 10^–5^ S/cm), which is insufficient for practical applications. Notably, ionic conductivity is highly sensitive to lithium salt concentration, exhibiting a nonmonotonic trend with changes in the lithium salt weight fraction. This pattern results from two competing effects: while increasing the lithium salt weight fraction initially raises ionic conductivity due to a higher free ion concentration, the polymer-ion interactions gradually reduce polymer dynamics as salt concentration rises, ultimately lowering ionic conductivity.

In PEO-based electrolytes the coordination of the cations by the oxygen atoms of the ethylene oxide (EO) monomers impart the alkali salt solubility and separation of the cations from the counteranions. However, too strong coordination leads to a decrease in cation mobility and corresponding ion conductivity which is detrimental for the practical application of the polymer electrolyte in battery applications. Switching from a Lithium salt to a Sodium salt in PEO-based electrolytes significantly impacts performance due to differences in ion size, coordination behavior, and interaction with the polymer. In PEO, due to its larger ion size, the coordination energy of Να^+^ with EO is lower than that of Li^+^ that in theory should allow faster ion transport and larger ion conductivity.^24,25^ Nevertheless, that is not what has been reported; the ion conductivity in PEO-Na^+^ based electrolytes has been reported to be lower than that of the corresponding PEO-Li^+^.^15,26−28^ The aforementioned data correspond to PEO molecular weight that is well above the molecular weight of entanglements (*M*_w_>*M*_e_), and ion hopping is mediated by ion hopping.

In this work, we aim to address several fundamental aspects of how cation chemistry affects the ionic conductivity of PEO-based electrolytes, which is crucial for unlocking their potential in designing polymer electrolytes with sufficiently high room-temperature conductivity. We utilize two different PEO samples: one with a molecular weight of 100 kg/mol, where ion transport primarily occurs through ion hopping, and another with a molecular weight of 0.35 kg/mol, where ion transport occurs via ion diffusion. These PEOs are blended with varying loadings of LiTFSI and NaTFSI salts. Our results demonstrate that in polymer electrolytes where ion transport occurs via hopping, smaller ions move faster (σ_LiTFSI_ > σ_NaTFSI_). However, in systems where ion diffusion is the primary transport mechanism, as with the low molecular weight PEO, larger ions exhibit higher mobility (σ_NaTFSI_ > σ_LiTFSI_). In the high molecular weight PEO, although Na^+^-ions exhibit weaker interactions with PEO at the monomer level, their larger size results in more Na^+^-EO contacts, leading to a higher coordination potential and slower ion hopping at room temperature. Conversely, in low molecular weight PEO, despite its smaller ionic radius, Li^+^ forms stronger interactions with the oligomeric PEO host. This results in a larger hydrodynamic radius and, consequently, slower diffusion and lower conductivity compared to Na^+^. Notably, at room temperature, the conductivity of PEO:NaTFSI is approximately σ_NaTFSI_ ≈ 4 × 10^–4^ S/cm, meeting the requirements for several practical applications. These findings suggest that for the development of Na-ion batteries using polymer electrolytes, low-molecular-weight PEO systems, where ion transport occurs predominantly via diffusion, hold significant promise.

Figure 1 presents the ionic conductivity of Li^+^-ions (black symbols) and Na^+^-ions (red symbols) as a function of temperature for PEO-based electrolytes with different molecular weights; PEO-0.35K (open symbols) and PEO-100K (filled symbols), at a fixed Li/EO ratio of *r* = 0.13. In the entangled PEO-100K, where ion transport primarily occurs via ion-hopping, the room temperature (RT) ionic conductivity for lithium ions, σ_LiTFSI_, is approximately 4 × 10^–5^ S/cm, about four times higher than that of sodium ions (σ_NaTFSI_ ≈ 1 × 10^–5^ S/cm). However, with increasing temperature, this gap progressively narrows, and both ion conductivities converge around 100 °C. In contrast, the low molecular weight, unentangled PEO-0.35K electrolytes exhibit the opposite behavior. Here, ion transport is dominated by ion diffusion rather than ion-hopping, and sodium ions display higher ion conductivity than lithium ions. At RT, σ_NaTFSI_ ≈ 3 × 10^–4^ S/cm, approximately an order of magnitude higher than that observed in entangled PEO-100K electrolytes. The significantly higher ionic conductivity in the unentangled PEO compared to the entangled PEO aligns with findings for Li-ion systems and highlights the inherently faster nature of ion diffusion relative to ion-hopping mechanisms.

**Figure 1:**
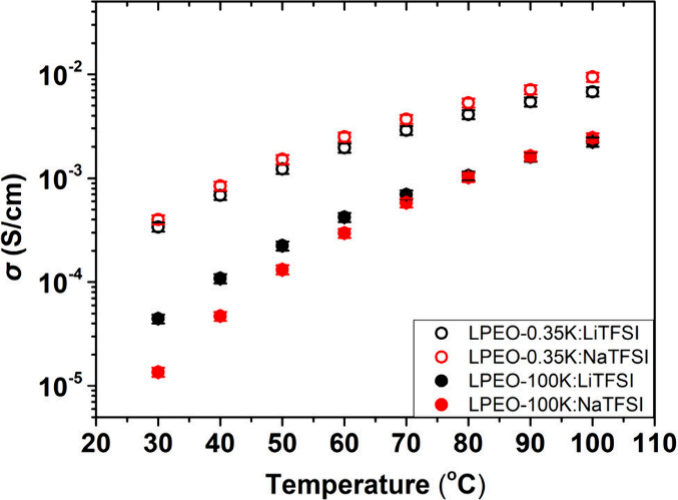
Li- and Na-ion conductivity (black and red symbols, respectively) as a function of temperature for PEO with a *M*_w_ = 100 kg/mol (PEO-100K, solid symbols) and 0.35 kg/mol (open symbols) at *r* = 0.13.

Figure 2 displays the ionic conductivity of lithium ions in PEO-based electrolytes with different molecular weights, specifically PEO-0.35K and PEO-100K, as a function of ion doping ratio *r* at 30 °C. Data are shown for both sodium (red symbols) and lithium (black symbols). Notably, near room temperature, which is relevant for battery operation, the conductivity trend depends on the *M*_w_ of the PEO matrix. For the low molecular weight PEO-0.35K electrolytes, sodium ions exhibit higher conductivity than lithium ions (σ_NaTFSI_ > σ_LiTFSI_). In contrast, in the high molecular weight PEO-100K electrolytes, σ_LiTFSI_ > σ_NaTFSI_^+^. In PEO-100K, there is a sharp drop in conductivity at low doping ratios *r* < 0.08, which is attributable to the crystallization of PEO (see Figures S1 and S2, Supporting Information), which reduces the amorphous content essential for ion transport. This structural change significantly impacts lithium ion conductivity in the entangled, high *M*_w_ matrix. Conversely, in the low molecular weight PEO-0.35K electrolytes, crystallization at low *r* occurs below RT. As a result, it does not impede transport at 30 °C., allowing sodium ion to maintain higher conductivity compared to lithium ions.

**Figure 2:**
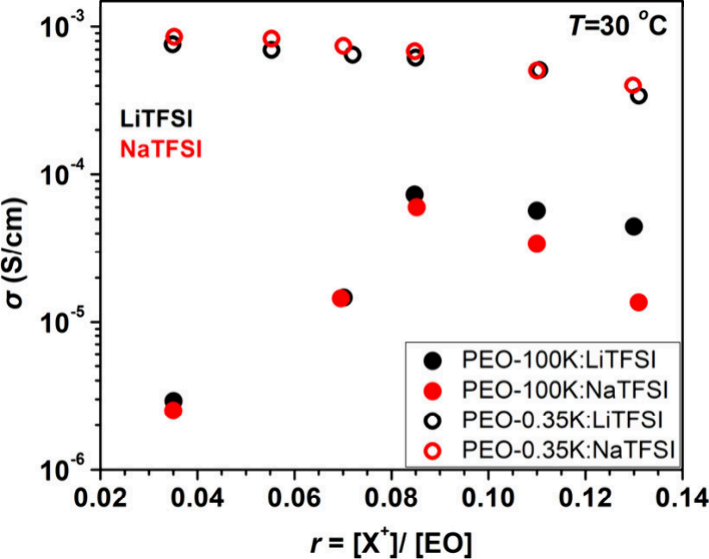
Ion-conductivity as a function of doping ratio *r* for PEG 0.35K (open symbols) and PEO 100K (filled symbols) for LiTFSI and NaTFSI (black and red symbols, respectively).

In PEO-100K based electrolytes, ion transport occurs primarily via ion-hopping between neighboring ether oxygen atoms, a process that is mediated by segmental dynamics. As variations in segmental dynamics are often reflected in the glass transition temperature, *T*_g_, DSC was utilized to measure the *T*_g_s of the polymer electrolytes under investigation in this work (Figure 3). It is immediately apparent that ion chemistry and degree of doping significantly impact *T*_g_ and that is also influenced by the molecular weight. Specifically, for entangled PEO electrolytes, *T*_g_ values of PEO-100K:NaTFSI electrolytes are consistently higher than those in PEO:LiTFSI electrolytes, indicating slower segmental dynamics in the presence of Na^+^. This trend aligns with the observed ion-conductivity behavior of these systems (solid symbols in Figures 1 and 2). However, for low *M*_w_, unentangled PEO, this trend are reversed; the *T*_g_ of PEO-0.35K:NaTFSI is lower than that in PEO-0.35K:LiTFSI.

**Figure 3:**
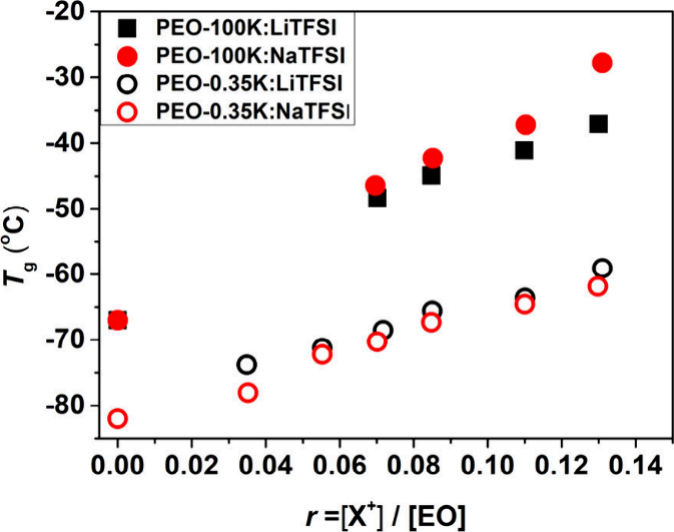
Effect of alkali salt chemistry (black and red symbols corresponds to LiTFSI and NaTFSI, respectively) on the glass transition temperature of PEO-0.35K and PEO-100K plots the glass transition temperature as a function of *r* (open and filled symbols, respectively).

To account for differences in polymer segmental dynamics (related to the glass transition temperatures shown in Figure 3), Figure 4 presents the ionic conductivities of PEO-100K:LiTFSI and PEO-100K:NaTFSI normalized to the *T*_g_ of each sample. The importance of cation chemistry is evident from the fact that the two data sets do not collapse together; at a given distance from *T*_g_ (where segmental dynamics of each sample are expected to be similar), PEO-100K:NaTFSI exhibits higher conductivity, opposite to the trend at the same absolute temperature in Figure 1. This behavior is attributed to the apparent higher activation energy for ion transport in PEO-100K:NaTFSI electrolytes being larger than that of PEO-100K:LiTFSI electrolytes; as evident by the slopes of the corresponding data in Figure 4.

**Figure 4:**
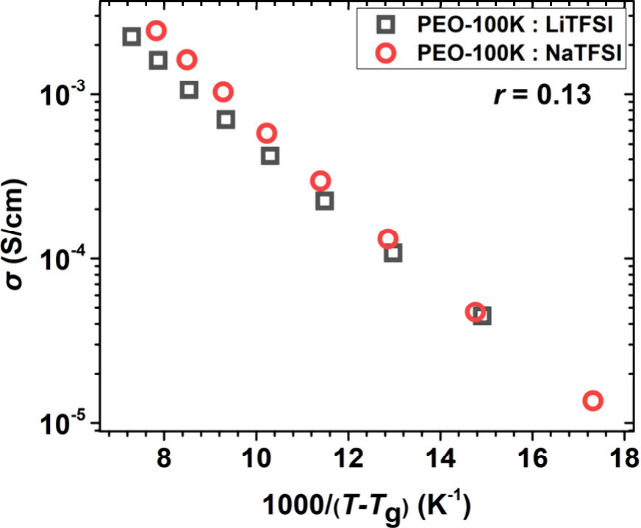
Ionic conductivity vs 1000/(*T* – *T*_g_) of the PEO-100K:LiTFSI (black opem squares) and PEO-100K:NaTFSI (open red cycles).

To further investigate the impact of ion chemistry on ion conductivity and *T*_g_ in entangled PEO-100K electrolytes, rheological measurements were conducted. Oscillatory frequency sweeps were performed across a wide temperature range, and the data were shifted using the time–temperature superposition (tTS) principle to create master curves. Figure 5 shows these master curves for PEO-100K:LiTFSI (black symbols) and PEO-100K:NaTFSI (red symbols) at *r* = 0.11. This particular *r* was selected as both PEO-100K:LiTFSI and 100K:NaTFSI are amorphous (see Figures S2 and S3 in the Supporting Information). The effectiveness of tTS was confirmed by the smooth van Gurp–Palmen plots (see Figure S4 in the Supporting Information). The PEO:NaTFSI electrolyte shows a narrower rubbery plateau in the intermediate frequency region compared to PEO-100K:LiTFSI electrolytes. This suggests that the larger Na^+^ ions reduce the number of effective PEO entanglements. This effect is likely due to the larger ionic radius of Na^+^, which limits polymer chain flexibility and ability to form entanglements. Consequently, a greater density of EO-Na^+^ contacts compared to EO-Li^+^, slows down segmental dynamics in NaTFSI containing electrolytes. In particular, Li^+^-ions have smaller ionic radius and form stronger individual EO-Li^+^ interactions at the monomer level, leading to higher association energy per contact compared to EO-Na^+^_._ Despite these stronger interactions, fewer EO-Li^+^ contacts that form per chain, results in a lower cumulative association energy (analogous to the product of EO-Li^+^ interactions times the number of EO-Li^+^ contacts) compared to Na^+^-based electrolytes and Li^+^ ions exhibit faster hopping dynamics across the PEO-100K matrix compared to Na^+^. In other words, our data indicate that while each EO-Li^+^ interaction is stronger, the reduced number of contacts per chain facilitates faster ion transport. In contrast, the higher number of EO-Na^+^ interactions per chain increases the cumulative association energy, leading to slower ion-hopping and segmental dynamics in Na^+^-containing systems.

**Figure 5:**
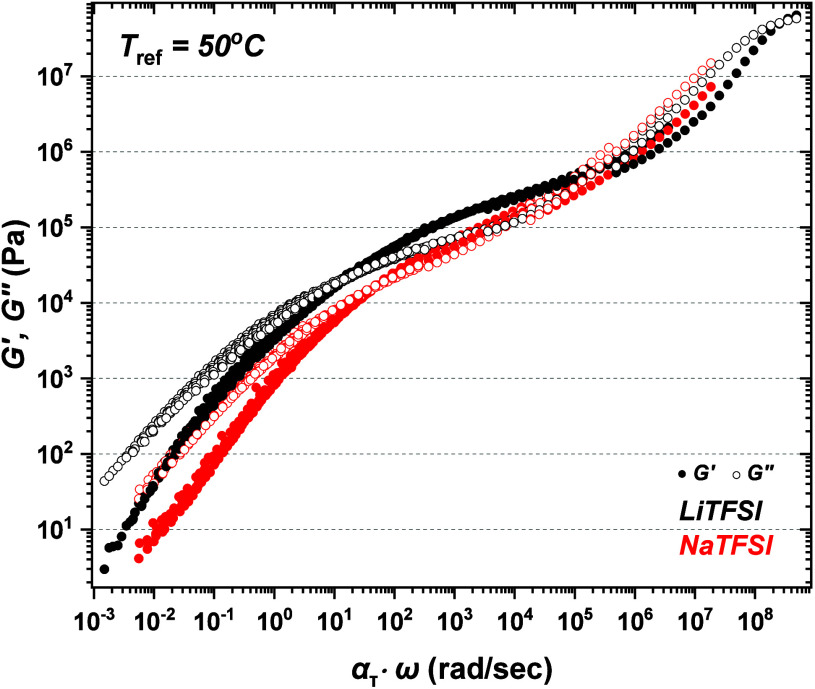
Master curves of PEO-100K:LIFSI (black symbols) and PEO-100K:NaTFSI (red symbols) generated using tTS that were horizontally shifted to a reference temperature of 50 °C. Solid symbols correspond to the storage modulus (*G*′) while open symbols correspond to loss modulus (*G*″).

As previously discussed, cation chemistry shows an opposite trend in low molecular weight, unentangled PEO systems compared with the high molecular weight, entangled PEO-100K system. In the low *M*_w_ system, larger Na^+^ cations show higher ionic conductivity than smaller Li^+^ cations, i.e., σ_NaTFSI_ > σ_LiTFSI_. This behavior is attributed to the stronger interactions of the Li^+^ ions with the oligomeric PEO chains due to the shorter ion radius resulting in stronger Li^+^-EO. The latter, increase the hydrodynamics radius and reduce in mobility; based on Stokes–Einstein relationship the diffusion coefficient (*D*) is inversely propotional to its hydrodynamics radius (*R*_h_), *D* ∝ *R*_h_^–1^. In this context, *R*_h_ refers to the effective size of the cation-oligomeric PEO complex moving through the oligomeric PEO matrix. Smaller cations, such as Li^+^, exhibit stronger interactions with the ether oxygen atoms (EO) in PEO due to their higher charge density and smaller ionic radius. These strong interactions lead to the formation of larger effective complexes (higher *R*_h_) as the Li^+^ ions are more tightly bound to multiple EO units. This trend has been reported in aqueous electrolytes in which smaller cations has a larger size in their hydrate state (Li^+^-H_2_O^δ−^ (3.82 Å) > Na^+^-H_2_O^δ−^ (3.58 Å)) and showed lower ion mobility (7.7 × 10^–2^ S/cm and 9.0 × 10^–2^ S/cm for Li- and Na-based aqueous electrolytes, respectively).^29,30^ Therefore, despite the smaller ionic size of Li^+^ compared to Na^+^, the stronger EO-Li^+^ interactions at the molecular level results in larger *R*_h_ compared to that of Na^+^, and consequently, Li^+^ ions diffuse more difficult through the PEO matrix resulting in σ_LiTFSI_ < σ_NaTFSI_.

As discussed above, in unentangled, low molecular weight PEO electrolytes, ion transport is primarily governed by ion diffusion, closely linked to the system’s viscosity. Rheological analysis of PEO-0.35K electrolytes shows a liquid-like Maxwell behavior, with the viscous modulus (*G*″) dominating the elastic modulus (*G*′), and frequency-dependent trends confirming this relationship (*G*″ ≫ *G*′ with *G*′ ∼ ω^2^ and *G*″ ∼ ω). Plotting the zero-shear viscosity (η_0_) against ionic conductivity (σ) for PEO:LiTFSI and PEO:NaTFSI electrolytes across various temperatures (Figure 6) reveals a clear overlap, indicating that ionic conductivity depends directly on viscosity. The cation size and interaction strength affect the viscosity, with Li^+^ ions forming stronger interactions with PEO chains, increasing local viscosity, and reducing ion mobility, while Na^+^ ions exhibit weaker interactions, leading to lower viscosity and enhanced mobility.

**Figure 6:**
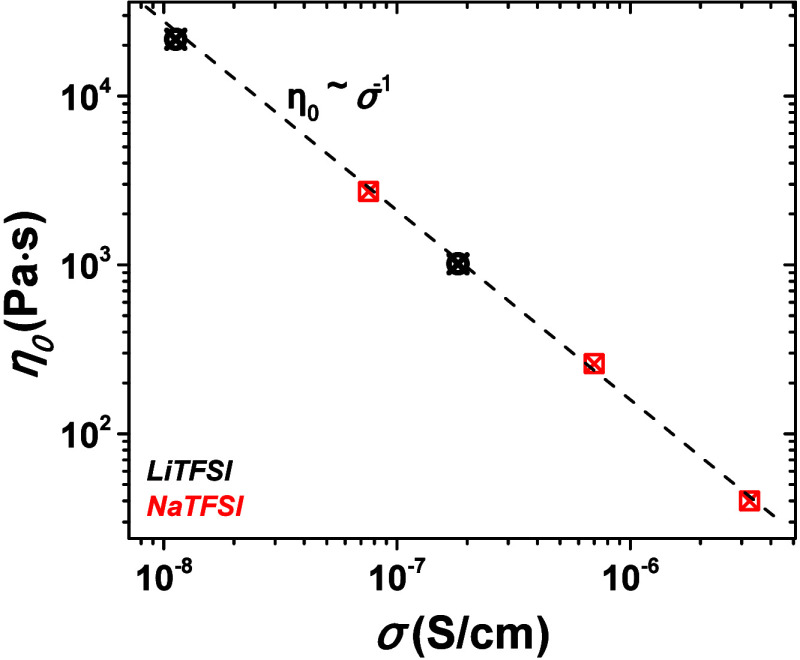
Zero-shear viscosity (η_0_) as a function of ion-conductivity for the PEO-0.35K:LiTFSI electrolytes (black symbols) and PEO-0.35K:NaTFSI electrolytes (red symbols). The black dotted line is the η_0_ ∼ σ^–1^.

In conclusion, we demonstrate a nontrivial interplay between the molecular weight of PEO, cation size, and the strength of cation-PEO interactions on the ionic conductivity of these electrolytes at room temperature. For entangled PEO systems (*M*_w_ > *M*_e_), PEO:NaTFSI exhibits lower ionic conductivity compared to PEO:LiTFSI, i.e., σ_NaTFSI_ < σ_LiTFSI_, whereas in unentangled PEO electrolytes (*M*_w_ < *M*_e_), the trend reverses, σ_NaTFSI_ > σ_LiTFSI_. This behavior arises because, in entangled PEO, the larger Na^+^ cations form more transient EO:Na^+^ contact, hindering cation hopping and reducing conductivity. In contrast, for unentangled PEO, where ion transport occurs via diffusion, the stronger EO:Li^+^ interactions lead to lower ionic conductivity compared to Na^+^ systems due to an increase in the hydrodynamics radius. These findings provide valuable insights into the design of more efficient polymer electrolytes, particularly for next-generation, beyond-lithium energy storage applications. The investigation of other cation chemistries, including both single- and multivalent ions,^31^ is part of an ongoing work.

## Supporting Information Available

Additional experimental details and data on materials, sample preparation, DSC, EIS and rheology on the PEO-based electrolytes (PDF)
