# Supplementary material for: Cation Chemistry and Molecular Weight Effects on the Ion Conductivity in PEO-based Electrolytes
Source: ACS Macro Lett. 2025 Feb 10;14(2):225–30. doi: 10.1021/acsmacrolett.4c00802 (PMC11841022; doi:10.1021/acsmacrolett.4c00802)
Supplement: Supplementary file 1 — mz4c00802_si_001.pdf [file mz4c00802_si_001.pdf]

SUPPORTING INFORMATION

Cation Chemistry and Molecular Weight Effects on the Ion Conductivity in PEO-based Electrolytes

Chrysostomos Papamichail,[1,2] Olympia Techlemtzi,[3] Georgia Nikolakakou,[2,3] Emmanouil Glynos,[1,2,*]

[1]Department of Materials Science and Engineering, University of Crete, P.O. Box 2208, 710 03, Heraklion, Crete, Greece

[2]Institute of Electronic Structure and Laser, Foundation for Research and Technology-Hellas, P.O. Box 1385, 711 10 Heraklion, Crete, Greece

[4]Department of Chemistry, University of Crete, P.O. Box 2208, 710 03 Heraklion, Crete, Greece

EXPERIMENTAL SECTION

MATERIALS

The materials used in this study include hydroxyl-terminated poly(ethylene oxide) (PEO) (Mv ~ 100 kg mol^{-1}, abbreviated as PEO 100k), mixed hydroxyl/methoxyl-terminated poly(ethylene glycol) (MethoxyPEG) (Mw = 350 g mol^{-1}, abbreviated as PEG 0.35k), lithium bis(trifluoromethanesulfonyl)imide (LiTFSI), and sodium bis(trifluoromethanesulfonyl)imide (NaTFSI, 97% purity). Chloroform and tetrahydrofuran (THF), both in anhydrous form and sealed under an inert atmosphere, were used to dissolve PEO 100k and PEG 0.35k, respectively. All materials were purchased from Sigma-Aldrich.

POLYMER ELECTROLYTE PREPARATION

The electrolyte blends were prepared and mixed in an argon-filled environment (MBRAUN glovebox) for at least one week. Before use, the salts were dried at 160°C under vacuum for 72 hours inside the glovebox. To dissolve mixtures involving PEG 0.35k, THF was added and stirred overnight to fully homogenize the XTFSI salt (X = Li^{+} or Na^{+}) and the dry polymer, without requiring thermal stimulation. For PEO 100k blends, chloroform was mixed with the polymer-salt solution and stirred overnight at 50°C on a hot plate until fully dissolved. Both series appeared visually transparent; however, the PEO 100k complexes were slightly hazy due to the polymer's nature. After stirring, the samples were dried at room temperature in the glovebox for approximately 48 hours. They were

then transferred to the glovebox antechamber and further dried under vacuum for 24 hours. To ensure complete removal of any residual solvent, the samples were finally heated at 100°C under vacuum on a hot plate inside the glovebox. The salt concentration of the electrolyte samples, defined as the molar ratio of cations to ethylene oxide units (EO), is given by: r = [X^+]/[EO], where X = Li^+ or Na^+. Samples were prepared at r = 0.035, 0.055, 0.07, 0.085, 0.10, and 0.13 for the PEG 0.35k series, and at r = 0.035, 0.07, 0.085, 0.10, and 0.13 for the PEO 100k series.

DIFFERENTIAL SCANNING CALORIMETRY (DSC)

The thermal properties were analyzed using differential scanning calorimetry (DSC) with a Discovery DSC 250 (TA Instruments). Measurements were carried out across a temperature range of -100°C to 110°C, applying a heating and cooling rate of 10°C/min. Data from the second and third heating cycles were used to determine the glass transition temperature (T_g) and the degree of crystallization (X_c). The Tg was identified from the inflection point of the sigmoidal exothermic step in the DSC heating curve. The degree of crystallization (Xc) was calculated by evaluating the area under the melting peak, which corresponds to the enthalpy change (ΔH) during the melting transition. This value was normalized to the PEO weight fraction in the sample and compared against the theoretical heat of fusion for 100% crystalline PEO (ΔH_cryst = 196.4 J/g)[1] using the formula

$$\chi_c = \frac{\Delta H}{\Delta H_{cryst}} \cdot 100\%$$

IONIC CONDUCTIVITY MEASUREMENTS

Ionic conductivity measurements were performed using a Bio-Logic SP300 potentiostat with two custom-made test cells. Each cell consisted of a pair of blocking stainless steel electrodes and a cylindrical Kentron 1000 PEEK spacer (Mitsubishi Chemical Advanced Materials). Complex impedance measurements were conducted inside the glovebox at selected temperatures. The cells were initially heated to 100°C and then cooled to 30°C, with measurements taken at 10°C intervals during the cooling process. A sinusoidal voltage of 50 mV was applied, and data were collected over a frequency range from 7 MHz to 1 Hz. All samples formed a semicircular curve, and the extrapolated intercept of the semicircle with the real resistance axis was measured as the characteristic resistance of the cell. The bulk resistance (R_b) could also be determined from the real part of the complex impedance at the frequency-independent plateau in the Bode plot, according to Equation 1 (both methods converged to the same conductivity values). In the latter, the conductivity (σ) was then calculated using the following equation:

$$\sigma = \frac{L}{R_b A} \qquad (2)$$

where L is the spacer thickness and A is the area of the electrode-sample interface. The equilibrium time at each temperature step was maintained at 30 minutes to ensure consistent measurements.

MECHANICAL PROPERTIES

Rheological measurements of the PEO 100k/XTFSI samples (X = Li^+ and Na^+) were conducted using an Advanced Rheometric Expansion System (ARES) equipped with a

2kFRT-N1 torque rebalance transducer (TA Instruments, USA). Measurements were performed in a parallel plate geometry with a plate diameter of 8 mm. The temperature-dependent behavior was studied over the range of 150°C to -30°C for PEO 100k/LiTFSI and 150°C to -20°C for PEO 100k/NaTFSI, using 10°C intervals. To ensure thermal equilibrium, samples were stabilized at each temperature for 20 minutes before measurements. The viscoelastic properties were measured using small-amplitude oscillatory shear (SAOS) tests within a frequency range of 10^{-2} to 10^{2} rad/s. To determine the linear viscoelastic regime, strain-sweep tests were initially conducted at a constant frequency—first at the maximum frequency (10^{2} rad/s) and, if necessary, at 10 rad/s and 1 rad/s to enhance the torque signal. Dynamic frequency sweep tests were then carried out over the 10^{-2} to 10^{2} rad/s range using the established strain amplitude.

DSC measurements

The cooling (right plots) and heating (left plots) DSC traces for the PEO-0.35K with LiTFSI (black lines) and NaTFSI (redlines) are shown in Figure S1. The blue lines corresponding to the pure, pristine LPEO-0.35K. The corresponding data for the PEO-100K are shown in Figure S2. As both cases the addition of LiTFSI and NaTFSI salt leads to the degrease of crystallinity and also shift the Tg of PEO to larger temperatures. The degree of crystallization as a function of doping is plotted Figure S3.

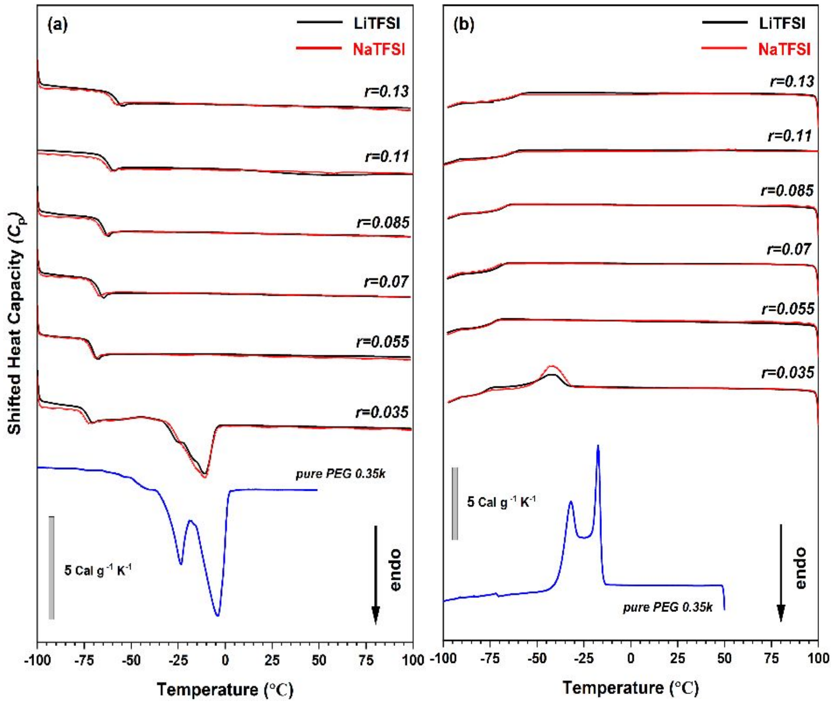

Figure S1: DSC thermograms of the PEG 0.35k : XTFSI electrolytes (X = Li^+ and Na^+, black and red symbols, respectively) during (a) heating and (b) cooling at a rate of 10^oC/min.

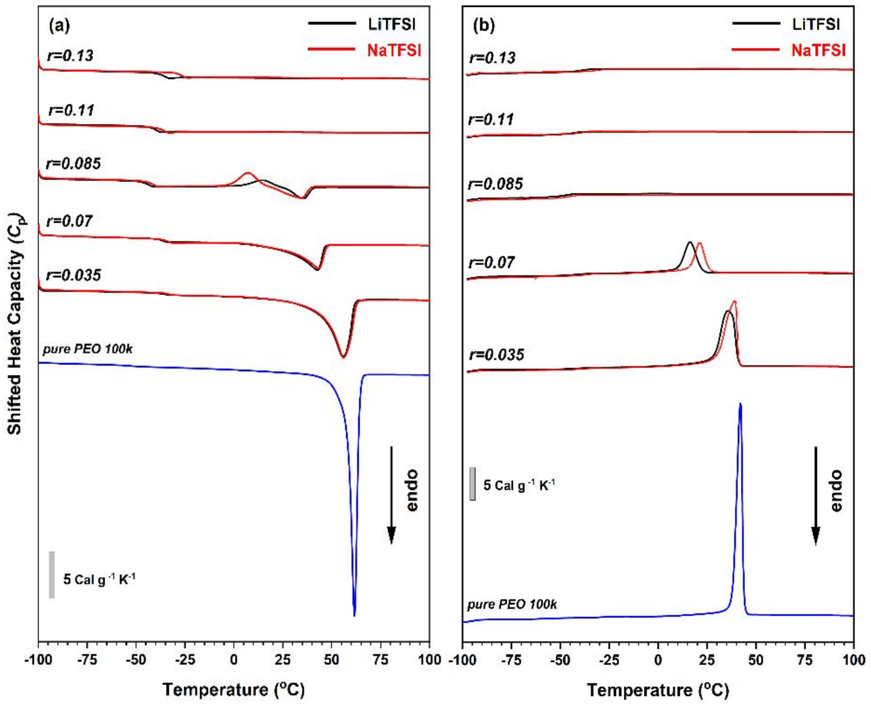

Figure S2: DSC thermograms of the PEG 100K : XTFSI electrolytes (X = Li^+ and Na^+, black and red symbols, respectively) during (a) heating and (b) cooling at a rate of 10°C/min.

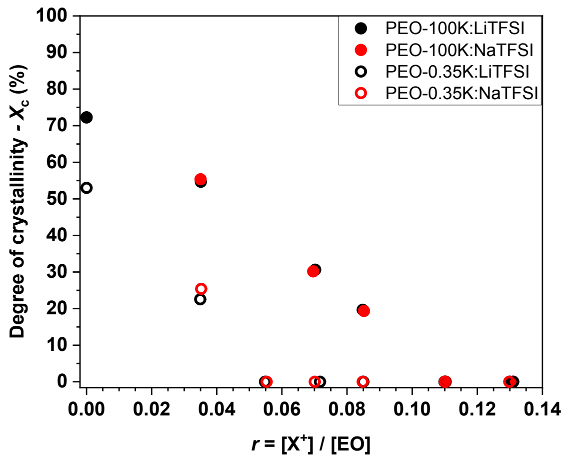

Figure S3: Effect of cation doping r = [X] / [EO] (where X: Li^+ or Na^+, black and red symbols, respectively) on the degree of crystallinity of PEO-0.35K (open symbols) and PEO-100K (solid symbols).

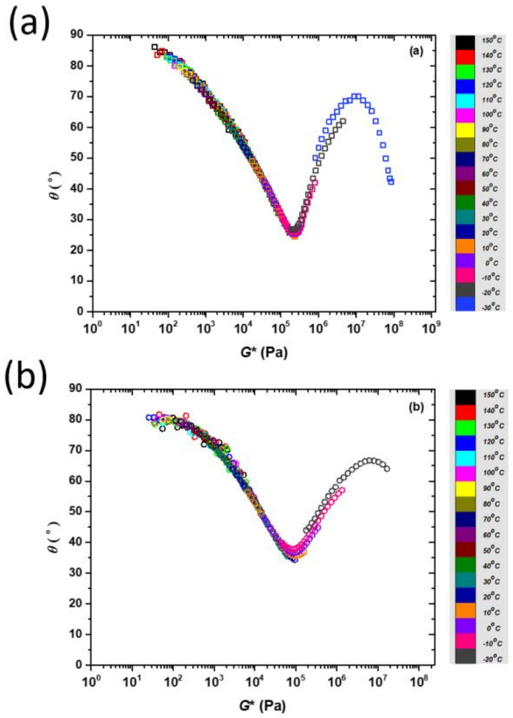

Figure S4: Van-Gurp – Palmen analysis of (a) LiTFSI:PEO-100K and (b) NaTFSI:PEO-100k at r=0.11.

REFERENCES

1. Dreezen, G.; Koch, M. H. J.; Reynaers, H.; Groeninckx, G. Miscible binary blends of poly(ethylene oxide) and an amorphous aromatic polyamide (Aramide 34I): crystallization, melting behavior and semi-crystalline morphology. *Polymer* **1999,** 40 (23), 6451-6463 DOI: https://doi.org/10.1016/S0032-3861(98)00849-0.

2. Agarwal, P.; Srivastava, S.; Archer, L. A. Thermal Jamming of a Colloidal Glass. *Physical Review Letters* **2011,** 107 (26), DOI: 10.1103/PhysRevLett.107.268302.

3. Agapov, A. L.; Sokolov, A. P. Decoupling Ionic Conductivity from Structural Relaxation: A Way to Solid Polymer Electrolytes? *Macromolecules* **2011,** 44 (11), 4410-4414 DOI: 10.1021/ma2001096.
